# Disseminated alveolar echinococcosis resembling metastatic malignancy: a case report

**DOI:** 10.1186/s13256-017-1279-2

**Published:** 2017-04-18

**Authors:** Laura Caire Nail, Ezequiel Rodríguez Reimundes, Christelle Weibel Galluzzo, Dan Lebowitz, Yasmine Lucile Ibrahim, Johannes Alexander Lobrinus, François Chappuis

**Affiliations:** 1 Service de médecine interne générale, Hôpitaux Universitaires de Genève, Rue Gabrielle-Perret-Gentil 4, 1205 Geneva, Switzerland; 2 Service de médecine tropicale et humanitaire, Hôpitaux Universitaires de Genève, Rue Gabrielle-Perret-Gentil 4, 1205 Geneva, Switzerland; 3 Service de pathologie clinique, Hôpitaux Universitaires de Genève, Rue Gabrielle-Perret-Gentil 4, 1205 Geneva, Switzerland

**Keywords:** Alveolar echinococcosis, *Echinococcus multilocularis*, Zoonosis, Malignant mimics

## Abstract

**Background:**

Alveolar echinococcosis is a potentially lethal zoonosis caused by larval forms of the tapeworm *Echinococcus multilocularis*.

Humans are aberrant intermediate hosts who become infected by ingestion of egg-contaminated food or water or via physical contact with domestic or wild animals that carry the parasite in their small intestine. In humans, the disease usually affects the liver and can spread to other organs causing metastatic infiltration. In this report, we describe an advanced presentation of human alveolar echinococcosis mimicking metastatic malignancy.

**Case presentation:**

A 62-year-old white woman was evaluated for fever, jaundice, and abdominal pain, associated with significant weight loss. She lived in a rural area in Switzerland and used to eat wild forest fruits and mushrooms. She owned cats that used to hunt rodents.

On physical examination, she appeared severely ill with cachexia, altered mental status, jaundice, and massive hepatomegaly. Laboratory tests showed cholestasis with preserved liver function.

An abdominal computed tomography scan showed an enlarged liver with a huge cystic mass in the right lobe extending into the left lobe, infiltrating her hepatic hilum, causing intrahepatic bile duct dilation and occlusion of her right portal vein. A chest computed tomography scan showed multiple calcified bilateral pulmonary nodules. Her clinical and radiological presentation resembled an advanced neoplastic disease. Serologic tests for *Echinococcus multilocularis* were positive.

The diagnosis of alveolar echinococcosis was established on her past history of exposure, imaging, and serology results.

**Conclusions:**

Clinical presentation and radiologic imaging findings of disseminated alveolar echinococcosis can mimic metastatic malignancy, and diagnosis can be challenging in atypically advanced cases. As the incidence of human alveolar echinococcosis appears to be increasing in Europe and Switzerland, physicians should be aware of alveolar echinococcosis, its epidemiology, and its clinical features.

## Background

Alveolar echinococcosis (AE) is a potentially lethal zoonosis of the northern hemisphere caused by larval forms (metacestodes) of the tapeworm *Echinococcus multilocularis*.

The parasite resides in the small intestine of the definitive hosts, either carnivorous wild animals, such as foxes, or domestic animals, such as dogs and cats, which excrete eggs in their stools. They become infected while eating contaminated wild rodents, the natural intermediate host. After ingestion by the intermediate host, the eggs hatch in the small intestine and release oncospheres that migrate to the tissues where they develop cysts. Humans are aberrant hosts that replace the natural intermediate host in the parasite life cycle. In humans, *E. multilocularis* cysts grow very slowly, resulting in an incubation period of 5 to 15 years. The disease usually affects the liver producing an alveolar-like pattern of microvesicles. The parasite can spread from the liver to other organs causing metastatic infiltration [1–4].

Humans are infected by ingestion of egg-contaminated food or water or via physical contact with domestic or wild animals that have eaten infected animals. The role of foxes in the zoonotic transmission of AE appears to be important, as demonstrated by the increase of AE incidence in humans and natural intermediate hosts following an increase in the population of foxes in some regions of Europe over the last 20 years [5].

## Case presentation

A 62-year-old white woman was evaluated in a Swiss regional hospital for fever, itching, jaundice, and abdominal pain, associated with significant weight loss (15% of total body weight in the last 3 months). Progressive right upper quadrant discomfort had been present for the last 5 years but she did not seek medical advice.

She had no past medical history and did not take any medication. She lived in a rural area in Switzerland and used to eat wild forest fruits and mushrooms. She owned cats that used to hunt rodents. She had no known underlying immunosuppression and declared no risk factors for human immunodeficiency virus (HIV) infection.

On physical examination, she appeared severely ill with cachexia, altered mental status, skin and mucous jaundice, and massive hepatomegaly. Laboratory tests showed lymphopenia (0.77 G/l; normal range 1 to 4.5) with normal eosinophil count, as well as cholestasis with preserved liver function: total bilirubin 135 μmol/L (reference value 7 to 25), direct bilirubin 64 μmol/L (0 to 10), aspartate aminotransferase 40 U/L (11 to 42), alanine aminotransferase 49 U/L (9 to 42), alkaline phosphatase 802 U/L (25 to 102), gamma glutamyl transferase 376 U/L (9 to 35), prothrombin time 43% (>70%), and factor V >100% (>70%). A HIV test was not done.

**Fig. 1:**
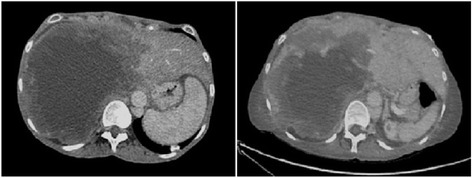
Abdominal computed tomography scan showing hepatomegaly with a 15 cm cystic mass in the right lobe with finely lobulated contours, and perilesional calcifications. The lesion extended into the left lobe of the liver, infiltrating the hepatic hilum, and provoking intrahepatic bile ducts dilatation, compression of the inferior cava vein and the left branch of the portal vein, and occlusion of the right portal vein

**Fig. 2:**
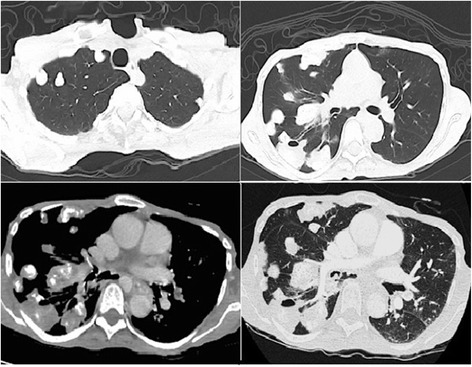
Thorax computed tomography scan showing multiple calcified pulmonary nodules up to 50 mm in diameter, some of which were cavitated

An abdominal computed tomography (CT) scan showed an enlarged liver with a 15 cm cystic mass in the right lobe with finely lobulated contours, and perilesional calcifications. The lesion extended into the left lobe, infiltrating her hepatic hilum, causing intrahepatic bile duct dilation, compression of her inferior vena cava and left branch of her portal vein, and occlusion of her right portal vein. Ascites was also present (Fig. 1). A chest CT scan showed multiple calcified pulmonary nodules up to 50 mm in diameter, some of which were cavitated (Fig. 2). An enzyme-linked immunosorbent assay (ELISA) and Western blot for *E. multilocularis* (EgP, EgHF and Em18 antigens) were positive. ELISA for *Echinococcus granulosus* was also positive but this was considered a cross-reaction. No other serological test was done. She was referred to our center for bile duct drainage, surgical evaluation, and initiation of anti-parasitic treatment.

**Fig. 3:**
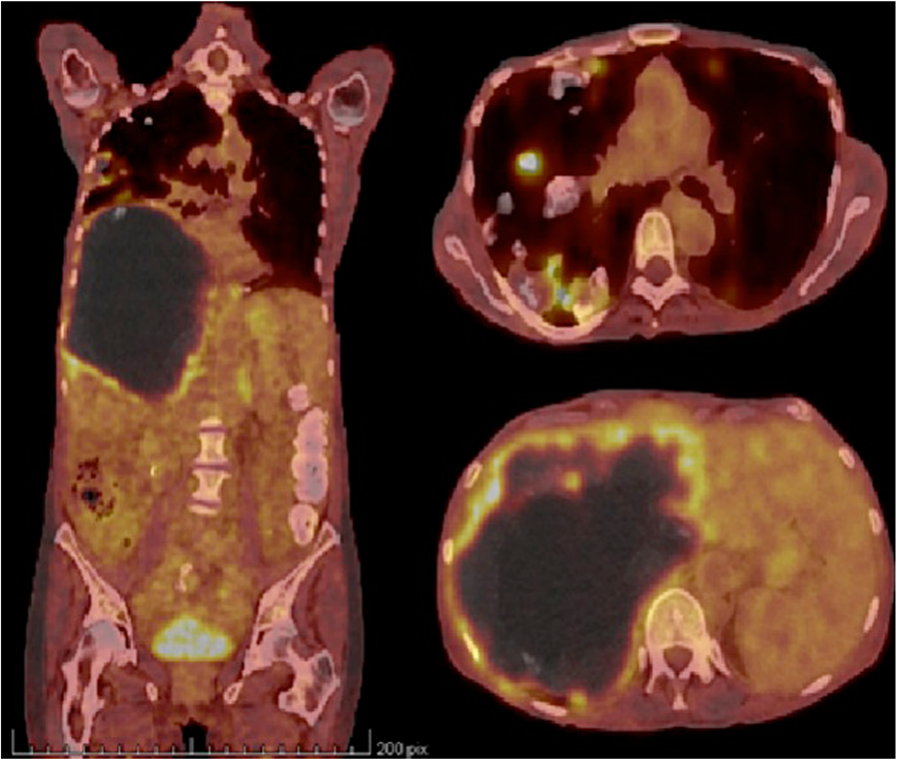
^18^F-fluorodeoxyglucose-positron emission tomography scanning showing multiple hypermetabolic lesions affecting both lungs, and a voluminous heterogeneous right hepatic lesion composed of tissue, liquid, and calcified elements surrounded by a ring of intense hypermetabolism

The diagnosis of AE was established on her past history of exposure, imaging, and serology results. Treatment with albendazole was started at a dose of 10 mg/kg per day with close follow-up of her liver function and blood count. The disease was classified as PNM stage IV (P4, N1, M1) according to the World Health Organization (WHO) classification. The extent of the disease and her bad general condition precluded liver transplantation. She benefited from an endoscopic drainage of her bile ducts with a bile stent placement leading to partial relief of pain and itching. Disease activity was assessed using an ^18^F-fluorodeoxyglucose-positron emission tomography (FDG-PET) scan, showing multiple hypermetabolic lesions affecting both lungs, and a voluminous heterogeneous right hepatic lesion composed of tissue, liquid, and calcified elements with intense hypermetabolism of its circumference (Fig. 3).

She developed agranulocytosis due to supratherapeutic plasma levels of albendazole sulfoxide and had severe oropharyngeal candidiasis. She then developed fever and abdominal pain. An abdominal CT scan showed gas bubbles within the cyst, evoking a bile duct bacterial infection.

A percutaneous drain was placed in the cyst. The procedure was complicated by *Escherichia coli* bacteremia associated with septic shock. She was admitted in the intensive care unit and her clinical course was further complicated by acute hepatic and kidney failure, encephalopathy, coagulopathy, and pulmonary embolism. Palliative care was provided and she died in the following days.

**Fig. 4:**
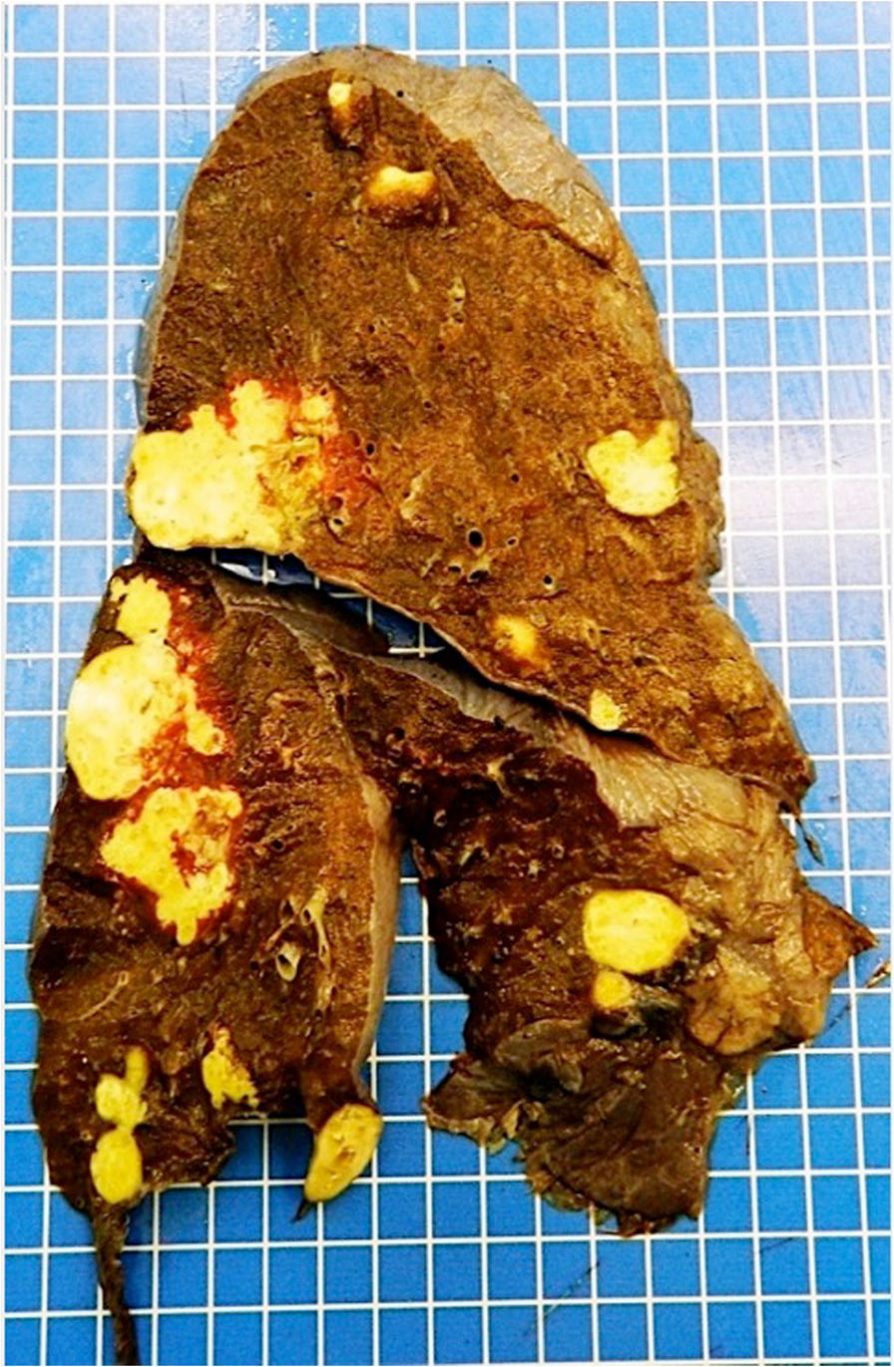
Sagittal section of the right lung showing cystic, necrotic, and focally calcified lesions of the three lobes measuring up to 4.5 cm in diameter

**Fig. 5:**
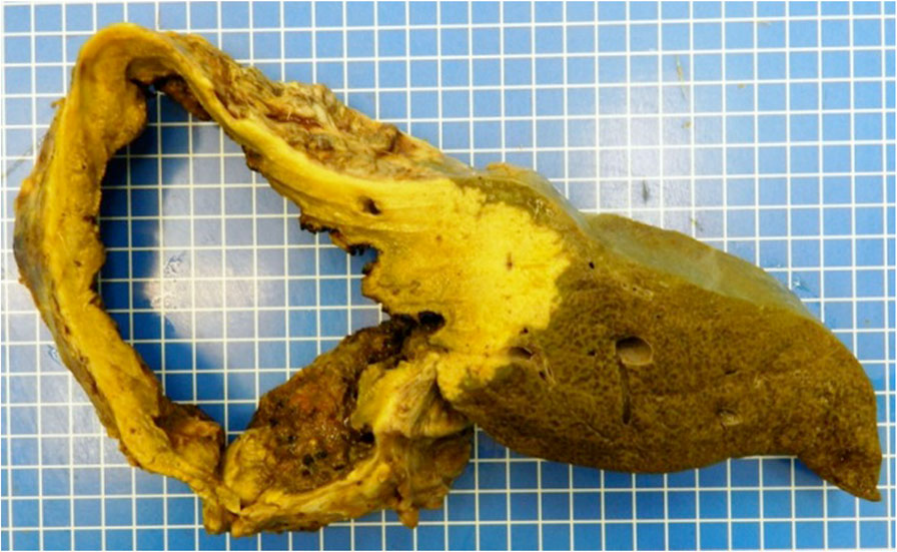
Liver section showing a cystic, partially necrotic lesion of the right lobe measuring 18.5 × 18 × 13 cm

An autopsy was performed. Macroscopic and microscopic examination confirmed AE and showed no evidence of neoplasia. At the pulmonary level, the autopsy found AE with cystic, necrotic, and focally calcified lesions of the three lobes of her right lung and the upper lobe of her left lung, measuring up to 4.5 cm in diameter (Fig. 4). Examination of her liver showed cystic AE partially necrotic in the right lobe measuring 18.5 × 18 × 13 cm (Fig. 5). No other organ involvement was identified at autopsy.

**Fig. 6:**
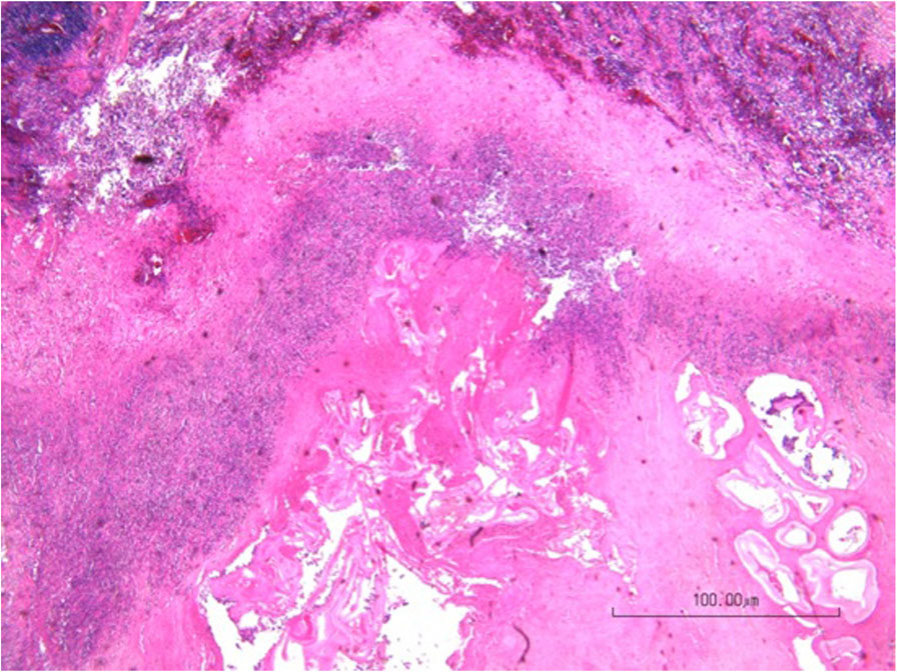
Histological examination of pulmonary cystic lesions (hematoxylin eosin) shows extensive fibrosis with inflammatory cells and necrosis surrounding alveolar echinococcosis cysts that contain laminated membranes

**Fig. 7:**
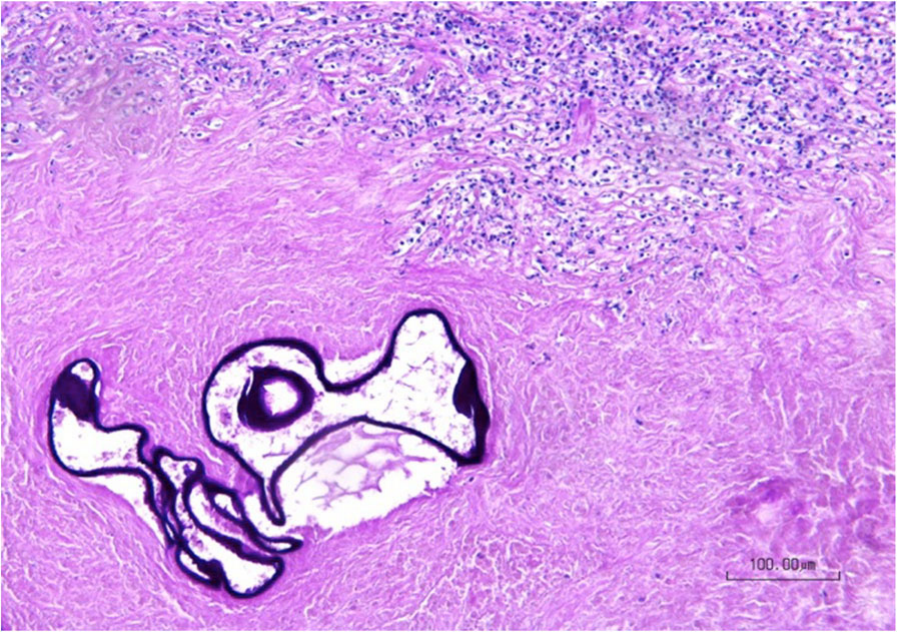
Histological examination of pulmonary cystic lesions shows laminated membranes in alveolar echinococcosis cysts delineated by periodic acid–Schiff stain

On histological examination of the pulmonary and hepatic lesions, irregular cysts containing periodic acid–Schiff (PAS)-positive laminated membranes were seen. These cysts were surrounded by an extensive peripheral fibrosis with fibroblasts, inflammatory cells, focal calcifications, and necrosis (Figs. 6, 7). Some of the cavitary pulmonary lesions were colonized by *Aspergillus* species.

## Discussion

This case illustrates the cancer-like presentation of *E. multilocularis* with infiltrative growth and metastatic spread.

In a case-control study performed in Germany the following risk factors for acquiring AE were identified: working as a farmer, living in a farmhouse, owning dogs that kill game, owning dogs and cats that roam outdoors unattended, collecting wood, chewing grass, living close to fields, walking in the woods for recreational reasons, growing leaf or root vegetables, and eating unwashed strawberries [6]. Some of these risk factors were present in our patient.

An initial asymptomatic incubation period of 5 to 15 years and a subsequent chronic course are typical of AE. *E. multilocularis* larvae proliferate like a slow-growing liver tumor, with a high mortality rate (about 90% at 10 years of diagnosis) in the absence of curative surgery and anti-helminthic treatment. Numerous local complications can occur, including biliary obstruction, cholangitis, sepsis, portal hypertension, and Budd–Chiari syndrome. Extrahepatic locations are rare [7].

Diagnosis of AE is based on clinical findings and epidemiological data, imaging studies, histopathology, nucleic acid detection, and serology. FDG-PET scanning demarcates areas of parasitic activity and is useful to assess the response to treatment. It is noteworthy that FDG-PET indicates suppressed inflammatory activity rather than parasite eradication [4]. In our patient, FDG-PET showed intense metabolic activity in pulmonary and hepatic lesions.

The WHO Informal Working Group on Echinococcosis (IWGE) PNM classification system, based on imaging findings, has been established to standardize diagnostic and therapeutic measures. It denotes the parasitic mass in the liver (P), the involvement of neighboring organs (N), and metastases (M), thus highlighting the malignant nature of AE.

Treatment should be planned within a multidisciplinary team that should include at least a surgeon, radiologist, hepatologist, and infectious-diseases physician with extensive experience in clinical parasitology. Radical surgery is the treatment of choice. However, surgery is reserved for early-stage disease when lesions can be completely resected with a safe (≥2 cm) margin of unaffected tissue and no distant metastases. Complete surgical resection of the parasite at an early stage of infection provides favorable prospects for cure, but since most cases are detected at an advanced stage, curative surgery can be performed in only 35% of patients. Non-radical liver surgery, previously regarded as beneficial for reducing the parasitic mass, does not appear to offer advantages over conservative treatment. Palliative surgery is almost always contraindicated [4].

Liver transplantation has been performed in patients with inoperable lesions and/or chronic liver failure. However, immunosuppression may favor growth of larval remnants and metastases [8, 9]. Our patient was neither eligible for radical resection nor for liver transplantation due to the disseminated disease and her altered general condition associated with incurable biliary tract infection.

Long-term benzimidazole (preferably with albendazole) treatment is mandatory in all inoperable patients as well as following radical surgery. Frequent adverse effects include alopecia, hepatotoxicity, and neutropenia, as observed in our patient. Screening for adverse reactions such as liver tests alterations and leukopenia is recommended every 2 weeks during the first 3 months of treatment, then monthly during the first year, then every 3 months [4]. Albendazole is metabolized in the liver to albendazole sulfoxide. Blood levels of this active metabolite should be monitored in all patients, especially in the case of hepatocellular dysfunction and/or cholestasis. Albendazole is parasitostatic and rarely eliminates *E. multilocularis.* Lifelong treatment is thus generally required to inhibit or at least suppress parasite growth in patients who cannot benefit from radical surgery. For the latter, albendazole treatment can be stopped after 2 years. Despite its limited efficacy, long-term albendazole therapy has improved the life expectancy of patients with AE to a near-normal level [10].

Patients undergoing radical surgery have a better outcome, whereas older patients have a poorer prognosis than younger patients [10]. One study showed that the presence of metastasis is not an independent prognostic factor for AE-related mortality [11]. Two recent studies performed in Switzerland and France showed a drastic improvement of AE prognosis in the last years [10, 11].

## Conclusions

Clinical presentation and radiologic imaging findings of disseminated AE can mimic metastatic malignancy, as described in our patient. As the incidence of human AE appears to be increasing in Europe and Switzerland [5, 12] and as diagnosis can be challenging in atypically advanced cases, physicians should be aware of AE, its epidemiology, and its clinical features. Treatment consists of chemotherapy with albendazole and/or hepatic surgery based on a multidisciplinary decision.

## Abbreviations

AE: Alveolar echinococcosis
CT: Computed tomography
ELISA: Enzyme-linked immunosorbent assay
FDG-PET: Fluorodeoxyglucose-positron emission tomography
IWGE: Informal Working Group on Echinococcosis
PAS: Periodic acid–Schiff
WHO: World Health Organization
